# The Mercurial Treatment of Tabes Dorsalis

**Published:** 1909-12

**Authors:** Maurice Faure

**Affiliations:** Formerly House Surgeon of the Paris Hospitals and Salpêtrière; Director of the Institute of La Malou (Hérault)


### THE MERCURIAL TREATMENT OF TABES DORSALIS.

BY

MAURICE FAURE, M.D.,

*Formerly House Surgeon of the Paris Hospitals and Salpêtrière; Director of the Institute of La Malou (Hérault).*

THE question of the mercurial treatment of tabes has been very often debated in congresses and also learned societies in France during recent years. Diametrically opposite opinions have been expressed with equal firmness. On the side of the syphilographers, Fournier, Gaucher and Hallopeau trace a very fine dividing-line between syphilitic spinal complications which may be treated successfully with mercury, and tabes, which may be a derivative of syphilis, but which is not, according to these people, a syphilitic sequel. Thus put, the question cannot be decided by clinical evidence. In fact, if a patient considered as tabetic is successfully treated by mercurials, one may suspect, of course, that the author of the observation made a mistake in diagnosis, and mistook an invalid suffering from cerebro-spinal syphilis for a tabetic.

Other syphiligraphers have given quite different opinions, but they are categorically the same. Leredde (who has been followed by a certain number of his contemporaries) affirmed that the tabetic lesions were just like the syphilitic (the cutaneous syphilides, for instance), susceptible of being treated successfully with mercury for the same reasons, and by the same way. When a tabetic has not improved by mercurial treatment, we must suppose that this treatment was not well applied, and was probably insufficient. Hence an entire change in the mode of the mercurial treatment is adopted, which, instead of being practised by means of injections or frictions with small doses, is carried on by subcutaneous injections of large doses. Was it necessary, then, if they had not until now obtained good results from mercurial treatment in the properly so-called tabes, to admit that it was because this treatment had been insufficient ? Would sufficient treatment really bring new results which would modify the pathogenic conception and the prognosis of tabes ?

It must be understood that a reply to these questions cannot be made " off-hand," and that the only way to solve the problems is to observe a series of tabetics treated by the new process and a series not so treated, these observations lasting several years. This is exactly what Pitres and Ballet counselled at the Toulouse Congress of Medicine, 1902, at the time when the first facts quoted by Leredde, Lemoine, etc., astonished the neurologists.

These observations have been made, and to-day, about six years after the beginning of this debate, we will try and see what the result is.

At first we meet with two precise and curious statements :—

(1) During these last six years all French neurologists have given their opinions repeatedly. Raymond (whose opinion is very much the same as that of Fournier, Gaucher and Hallopeau) thinks that syphilitic meningo-medullary lesions, capable of being successfully treated with mercurial treatment, are often very much like tabes, at least during their primary stages. This tends to explain, from his point of view, the fact that people are able sometimes, in invalids considered as tabetics, to obtain by mercurial treatment the breaking-up, or even the retrogression, of important symptoms. Pitres, G. Ballet, P. Marie, although not declaring themselves as plainly as the other authors, seem to believe that this treatment is of no use for true tabetic lesions. Jeoffroy and Brissaud expressly deny any sort of improvement brought about by hydrargyrum treatment during the course of tabes. In spite of these divergences of opinion, all (Dupré, etc.) are agreed on the following points : (1) Any intense mercurial treatment, such as that which was extolled by Leredde, may be dangerous to tabetics ; (2) it seems to be useless. One French neurologist only, Babinski, is of quite an opposite opinion. Before Leredde had formed his opinion, which was so much talked of, Babinski had practised mercurial treatment of tabetics in a methodical way, and declared that he had obtained good results.

(2) In spite of the almost complete agreement of the principal neurologists against the opinion adopted by Babinski, Leredde and Lemoine, *their opinion has prevailed and gained ground from year to year, so much so, that now you can hardly see a tabetic not adopting mercurial treatment.* At the time when Leredde made his first publication, one could be certain that the tabetics attempting a serious mercurial treatment constituted the smallest exception. The intervention of Leredde and Lemoine, and Babinski's authority, created a prolonged sensation, which gave a new direction to tabetic therapeutics.

Therefore the question is plainly put and resolved in two different ways : on one hand by the authorities in neurology, on the other hand by medical men in general and invalids.

Let us examine the particulars of the problem, and see, if possible, where the disagreement lies.

We have to distinguish at first two different categories of tabetics. In the first one the tabetic, generally observed in the hospitals, is already afflicted with chronic maladies, complicated in different ways. It does not matter whether his former lesions were or were not syphilitic, since there only exist in his marrow extensive and old sclerous scars, upon which one could not demonstrate that mercurial treatment might have a hold, even if these scars were the results of purely syphilitic lesions. Mercurial treatment is unable to make the scar of a cutaneous gumma disappear; but in lesions not cutaneous, it may be well understood that irreparable sclerous scars may be left in the marrow. This scar has cut some cord, interrupted routes of communication, hindered sensations from reaching the centres which have to perceive and arrange them in order; in short, it has badly injured the sensory conducting functions of the region.

On the other hand, disturbances of the bladder, intestines and lungs, the consequences of this spinal lesion, bring about a stasis of the secretions into these organs, and secondary infection. These affections injure the general health, and at last the patient, afflicted with a sclerogenous process in his marrow, is generally attacked by analogous processes in other viscera—liver, kidney, etc. Then the depurative functions easily become incapable of doing their work, and it only needs the usual resort to analgesic poisons (such as morphine, heroin, antipyrin, pyramidon, etc.) to add to this insufficiency of action, so that the whole organism is not long in becoming permanently poisoned. Injured sensori-motor functions, insufficiency of intestinal, pulmonary and urinary elimination, infection of the cavities, fever, medicinal poisoning, such is the balance-sheet of the ordinary tabetic's morbid state. We shall call him the "*matured tabetic.*"

In opposition to this type of classical tabes is the patient who comes, in the majority of cases, to the physician's private consulting-room. Let us examine him. He has no more than Argyll-Robertson's sign, abolition of the patellar reflex, a little uncertainty when walking in the darkness, a few sharp pains, slight hindrance of micturition, sometimes a part only of these small defects. Very often, at such a time, a diagnosis is not even made ; the medical man hesitates before pronouncing a judgment he thinks to be inexorable. No doubt amongst the subjects afflicted with diplopia observed and attended by Fournier (the majority of whom did not become confirmed tabetics) a large number were in this category. What lesions do exist among those patients ? It is well known how few are the autopsies of " tabes incipiens," and how much the early details of tabetic lesions is still discussed. Numerous hypotheses are presented, but they are only hypotheses precisely for want of autopsies of tabes incipiens. We may be allowed, then, to suppose that now there is neither sclerosis nor incurable scar ; there may be, perhaps, the meningo-radicular processes pointed out by Nageotte, and described also by Sicard, perhaps an embryonal pre-sclerous infiltration of the posterior radicular co-ordinating fibres (*i.e.* lesions which can be cured quite easily), such being, probably, all that would be met with. But one has no fear in saying and repeating that often five, ten, fifteen, twenty years elapse before a patient showing these small symptoms finds the pronounced evidences of advancing tabes. Some invalids remain in this premonitory state during their whole lives ; or, according to an expression of Fournier, in this " ante-chamber of tabes."

To apply an intense mercurial treatment to the first or the second of these maladies, to the matured or to the " recent tabetic," is evidently quite a different matter. But during recent years the experiments made by neurologists have been carried on principally amongst tabetics of the first category. Therefore the results of mercurial treatment in these cases have been of little worth, and even sometimes bad. This was because the lesions were irreparable, because many symptoms depended on superadded lesions or complications upon which mercurial treatment had no effect, because among some of those patients already affected or in a state of permanent auto-intoxication the mercury was but one poison more added to many others.

On the other hand, the protagonists of mercurial treatment have not said that it must be the *only* treatment of tabes, and that it was by itself *alone* the only therapeutic treatment.

Between two tabetics—the one who is going on with his usual life, overworked with moral and material difficulties, sleeplessness, faults of alimentation and hygiene, frequent medicinal intoxication (morphine, antipyrin, pyramidon, heroin, etc.), were resulting from the overworking of an organism whose equilibrium is worn out; the other of the same age and condition, showing the same lesions, who as soon as the diagnosis is settled abandons any work, modifies his hygiene from top to bottom, sleeps, eats, does not take any exercise or rest but those prescribed—I say that between these two patients no therapeutic comparison is possible, were they both submitted to identical mercurial treatment. Even for the most notoriously syphilitic tabetic syphilis is far from being the one origin of all the symptoms of tabes. Some appear under the influence of an intercurrent disease (influenza, for instance), others from overwork, others are brought on by alcohol, overfeeding, etc.; in short, the simplest kind of tabes is still more complex than a cutaneous syphilide, and one is not able to satisfy oneself as to its treatment by the same reasoning.

Thus the disagreement between both the opinions quoted above occurs from the following considerations: (1) Leredde's affirmations were too categoric and simple, the subject being much more complex than he said; (2) patients described by believers in the mercurial treatment of tabes and those described by neurologists are not the same in the majority of cases.

How are we to understand nowadays the prognosis and treatment of tabes, and especially the mercurial treatment? Firstly, the diagnosis of tabes must be made precisely, and as near as possible at the beginning of the affection; no more spurious rheumatism, under which the physician too often has attempted to conceal from the patient the real meaning of the lightning pains, in order to spare him or his family the anguish which accompanies an outspoken and unexpected diagnosis of tabes; there should be no more hesitation for a period, during which the physician avoids accepting a responsibility which frightens him. For the tabetic, as for the tuberculous, one should make the diagnosis very early, and not hesitate, if necessary, to shock the family and the patient. One must admit that there is tabes as soon as the Argyll-Robertson and Romberg's signs appear, as soon as the first sharp-shooting pains, as soon as the first vesical disturbances, are noticed, just as you would affirm there is tuberculosis as soon as there is increase of resistance to percussion, the early differences of auscultation pointed out by Grancher, the commencement of hæmoptysis, however slight. To wait for the important signs of tabes in order to strengthen one's diagnosis, would be like waiting in a case of tuberculosis for the appearance of a subclavicular cavity. In both cases, as soon as the diagnosis is made, you must warn the invalid of the danger which threatens him, specifying plainly that the symptoms are but a warning, and that he can avoid after-effects if he is careful ; because, considered in this way, *tabes is curable.*

Tabes is curable, at least clinically, because there exist tabetics who have recovered ; but it is necessary to understand each other over the meaning of the word " recovered." These invalids may retain a few slight symptoms, they may keep some marks in their nervous centres, they may even have a relapse ; but in dealing with the old and classical comprehension of the word tabes (" *progressive locomotor ataxy* "), these points do not prevent that the invalid who for thirty years has had but a few attacks of sharp-shooting pains, abolition of patellar reflex and Argyll-Robertson's sign, may be practically considered as cured. Does not a tuberculous patient, when cured, retain some pulmonary traces of his lesion, some respiratory or circulatory symptom resulting from it ?

But if cases of curable tabes exist, if an early diagnosis and well-managed treatment check the progress formerly considered inexorable, and even give retrogressions, what is the part played by mercurial treatment ?

Cases of benign tabes become more and more frequent ; arrest of tabes is the rule, progress the exception ; amongst established symptoms some retrogress. At the moment of the set-back to progress, symptoms in a fair way to becoming established may be stopped. At last tabes, being checked, may remain so indefinitely, and the relapses, the halts, which were formerly the rule, may be prevented or may he accounted for by complications due to drugs.

Mercurial treatment better followed, longer and more generalised, is one of the causes, and probably the initial cause, of the transformation of the prognosis of tabes. *It is generally amongst patients who have followed, or will follow, a methodical and prolonged mercurial treatment that the evolution of the disease remains benign,* provided that they do not ignore other therapeutical rules, and that diagnosis is, and has been, made early, and treatment commenced in good time. It is amongst patients badly treated, or not cared for at all (who were formerly the rule), that the evolution of the disease remains progressive ; but evidently this rule admits of exceptions and corrections in one or the other of both its parts, as dogmatic affirmations in medicine always do.

We now approach the question, How is the mercurial treatment to be performed ?

We answer, as much as possible by injections of soluble salts, which permit a stricter surveillance of individual toleration. Calomel and grey oil ought to be reserved for strong and robust patients, and constitute for these cases the best procedure. For other patients the use of these two substances may be attended with fatigue, and sometimes with an increase of certain tabetic symptoms ; hence a difficulty arises in making the medication acceptable, and hence the complaints which are heard against it. Two or three centigrams of mercury benzoate or biniodide daily is a sufficient dose in the majority of cases. One should discontinue the injections in every case where the patient feels indisposed ; but if the salt is soluble, uneasiness will disappear with the elimination of the drug in one or two days.[1] In short,

[1] 1 to $1\frac{1}{2}$ centigram of mercury (metal) daily constitutes a normal dose and a good standard of reference. One centigram of mercury (metal) corresponds to—

| | |
|---|---|
| Calomel .. .. .. .. .. | 1.177 cg, |
| Mercury bichloride .. .. .. | 1.335 ,, |
| Mercury benzoate .. .. .. | 2.200 ,, |
| Mercury biniodide .. .. .. | 2.270 ,, |
| Hermophenyl .. .. .. .. | 2.500 ,, |

For the grey oil formulas are variable, Easily-managed grey oils exist, containing 10 centigrams of mercury (metal) to each cubic centimeter.

a repeated examination of the general and local state will give indications and permit one to graduate the mercurial treatment in every case, in order to make it as strong as possible, and, at the same time, without intoxicating the patient. Amongst invalids whose general state requires exceptional care the hydrargyrum cure may be made with smaller doses, and associated with or followed by an arsenical cure. Products of the hermophenyl series, such as hydrargol, levurargyrum, enesol, are generally well received. But some competent chemists regard their mercurial effects as uncertain. Yet I think I have obtained good results from them, and I think that one ought to give them to patients who cannot take the ordinary salts.

To sum up, one should prescribe calomel and grey oil when one may or ought to do it ; mercury benzoate and biniodide usually ; hermophenyl, etc, when the patient's sensitiveness demands benign therapeutics. Such is what seems to me should be the general routine. Large doses—more than one centigram of mercury (metal) by day—can be taken by many invalids, provided that its use is not extended for too long a time. When giving them, however, you will recollect that if you surpass the patient's toleration even slightly, and if you provoke in a nervous invalid toxic troubles or even mental ones, you put his organism out of order, and you aggravate his nervous symptoms, which aggravation may be sometimes lasting. In so doing you run counter to the object you have in view, turning the patient against the mercurial treatment, and putting obstacles in the way of its success. A series of from fifteen to thirty centigrams of mercury (metal), renewed about four times yearly, may be given in order to complete an average of one gram in the year. Theoretically, one centigram of mercury (metal) daily may serve as a standard, for the whole of a series, for ordinary patients. It will be recollected that the intensity of the treatment does not result either from the quantity of a daily dose, or from the nature of the salt employed, but from the whole dose of the series. Consequently, the series will become shorter as the daily dose becomes bigger.

In concluding, I wish to repeat that mercury is one of the elements of the tabetic's treatment, but not all. In consequence, the tabetic must continue to carry out all the other details of hygiene, general supervision, and physical therapeutics for which experience has shown the necessity. Success can only be secured by this means.

---

### "VIS MEDICATRIX NATURÆ."

